# The Contribution of Social Behaviour to the Transmission of Influenza A in a Human Population

**DOI:** 10.1371/journal.ppat.1004206

**Published:** 2014-06-26

**Authors:** Adam J. Kucharski, Kin O. Kwok, Vivian W. I. Wei, Benjamin J. Cowling, Jonathan M. Read, Justin Lessler, Derek A. Cummings, Steven Riley

**Affiliations:** 1 Department of Infectious Disease Epidemiology, London School of Hygiene & Tropical Medicine, London, United Kingdom; 2 MRC Centre for Outbreak Analysis and Modelling, Department of Infectious Disease Epidemiology, School of Public Health, Imperial College London, London, United Kingdom; 3 School of Public Health, Li Ka Shing Faculty of Medicine, The University of Hong Kong, Hong Kong; 4 Department of Epidemiology and Population Health, Institute of Infection and Global Health, Faculty of Health and Life Sciences, University of Liverpool, Liverpool, United Kingdom; 5 Department of Epidemiology, Johns Hopkins Bloomberg School of Public Health, Johns Hopkins University, Baltimore, Maryland, United States of America; New York University, United States of America

## Abstract

Variability in the risk of transmission for respiratory pathogens can result from several factors, including the intrinsic properties of the pathogen, the immune state of the host and the host's behaviour. It has been proposed that self-reported social mixing patterns can explain the behavioural component of this variability, with simulated intervention studies based on these data used routinely to inform public health policy. However, in the absence of robust studies with biological endpoints for individuals, it is unclear how age and social behaviour contribute to infection risk. To examine how the structure and nature of social contacts influenced infection risk over the course of a single epidemic, we designed a flexible disease modelling framework: the population was divided into a series of increasingly detailed age and social contact classes, with the transmissibility of each age-contact class determined by the average contacts of that class. Fitting the models to serologically confirmed infection data from the 2009 Hong Kong influenza A/H1N1p pandemic, we found that an individual's risk of infection was influenced strongly by the average reported social mixing behaviour of their age group, rather than by their personal reported contacts. We also identified the resolution of social mixing that shaped transmission: epidemic dynamics were driven by intense contacts between children, a post-childhood drop in risky contacts and a subsequent rise in contacts for individuals aged 35–50. Our results demonstrate that self-reported social contact surveys can account for age-associated heterogeneity in the transmission of a respiratory pathogen in humans, and show robustly how these individual-level behaviours manifest themselves through assortative age groups. Our results suggest it is possible to profile the social structure of different populations and to use these aggregated data to predict their inherent transmission potential.

## Author Summary

For infections such as influenza, there are several aspects to the transmission process, including the properties of the pathogen itself, the host immune system and host behaviour. Although it has been proposed that self-reported social mixing patterns can be used to explain the behavioural component of infection – and mathematical modelling studies based on reported social contacts are used routinely to inform health policy – it is not clear how these contacts contribute to individual- and group-level infection risk. By analysing the relationship between social contacts and infection patterns during the 2009 Hong Kong influenza pandemic, we show that infection risk was strongly influenced by the average reported social mixing behaviour of an individual's age group, rather than by their personal reported contacts. We also demonstrate how social contact surveys can be combined with mathematical models to create useful tools with which to study respiratory infections in humans. This should make it possible to predict how the impact of interventions will vary from one population to the next based on their contacts and, potentially, to explain differences in infection attack rates between groups with different mixing behaviours.

## Introduction

For directly transmitted respiratory pathogens such as influenza, an individual's risk of infection depends on several factors. As well as host physiology and the immune system changing naturally with age, previous exposure to related pathogens can affect the outcome of subsequent exposures [1], [2]. In addition, infection risk depends on behaviour that generates potentially infectious contacts [3], [4]. One way to measure such behaviour is through surveys of self-reported social contact patterns [4]–[8].

Mechanistic models incorporating data on self-reported contacts are being used increasingly frequently to examine the effect of social mixing on disease dynamics [8]–[11] and to assess potential control measures [12]–[20]. In these models, populations are structured by age, with the force of infection for a specific age group depending on their reported contacts with other ages [3]. Although there is some statistical evidence from age-aggregated cross-sectional serological data that such models capture infection risk [8], [11], [21], it is not conclusive. Further, it is not known what resolution of age-structured model – both in terms of number and size of age groups – reproduces observed attack rates best.

**Figure 1:**
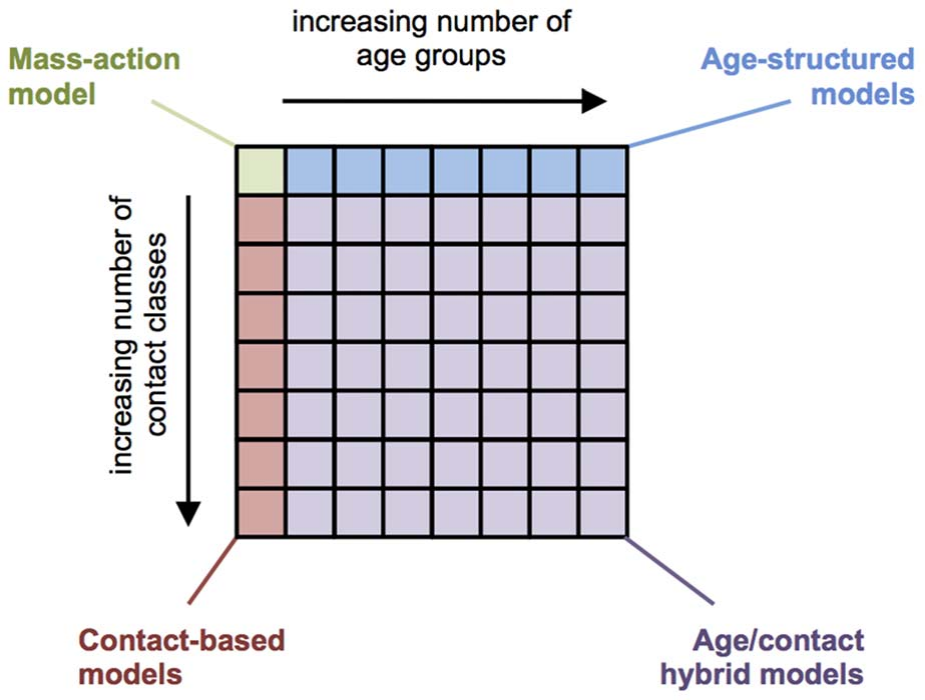
Schematic of model framework. By dividing the population into different numbers of age groups and contact classes, it was possible to recreate a number of commonly used model structures. If only one age groups and one contact classes were included, the framework produced a simple mass-action model, in which all individuals had the same risk of infection. When there was only one contact class in each age group, we obtained an age-structured model. Alternatively, when only one age group was used, risk of infection depended only on the contact class an individual was in.

Here, we report a comparison of alternate hypotheses about how age and self-reported social contacts influence risk of infection. We developed a flexible model framework that could incorporate both age and contact behaviour. The population was divided into increasingly finely resolved age and contact classes, with the transmission rate from one class to another proportional to reported contacts between those classes. Our formulation generalised a number of commonly used transmission models (Figure 1): by varying the number of age groups and contact classes, we could implement a simple mass-action model, an age-structured model [8]–[20], or a model in which individuals were structured only by their number of contacts [7], [22], [23]. Using data from a 2009/10 survey in Hong Kong [24], which included both reported social contacts and biologically confirmed infection status, we first explored how different model structures influenced patterns of infection. Next, we assessed to the extent to which each model captured observed attack rates, and established the structure and nature of social contacts that best explained influenza infection risk. Finally, we used these results to identify the resolution of social mixing that likely shaped influenza A/H1N1p transmission in 2009.

## Results

**Figure 2:**
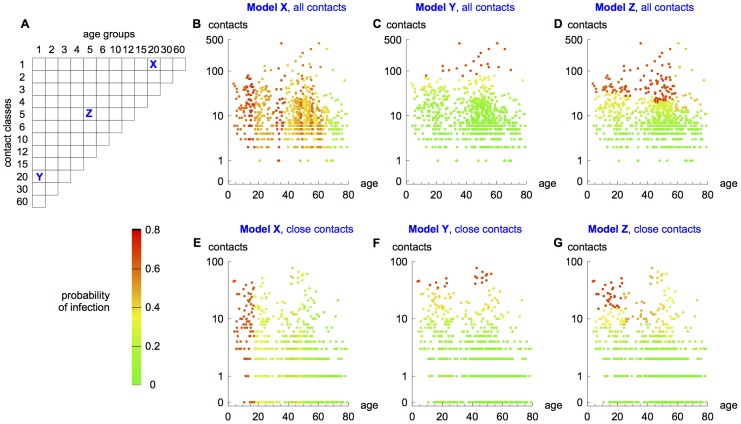
Risk of infection in different models. (A) Possible model structures. Given the size of the Hong Kong dataset, the maximum possible number of age and/or contact groups in a particular model was limited 60. (B) and (E) Results from model X, which has 20 age groups, each containing one contact class. Each point represents one of the 762 individuals surveyed, with position based on reported age and total number of contacts, and colour showing risk of infection predicted by the model. (C) and (F) Results from model Y (1 age group with 20 contact classes). (D) and (G) Results from model Z (5 age groups, each with 5 contact classes). Models are either based on all reported contacts (B, C and D), or close contacts only (E, F and G). *R_0_* = 1.5.

First we explored the theoretical potential of age and social behaviour to affect the infection attack rate in different population subgroups (Figure 2A). Incorporating the Hong Kong contact data, but not yet fitting to serological data, our model framework could produce a number of different patterns for the risk of infection. Figures 2B–G show the predicted risk of infection in different models compared with reported age and number of contacts for each of the 762 individuals sampled in the survey. If transmission was based on reported interactions between age groups, an individual's risk of infection was dependent solely on their age. Thus we obtained vertical bands in Figures 2B and 2E. It is noticeable that when close contacts were used, there was a much higher relative risk in school-aged individuals compared with older groups (Figure 2E). If we assumed transmission was dependent on reported contacts rather than on age, we see the opposite pattern: risk of infection fell into horizontal bands (Figures 2C and 2F). Based on existing literature [7], [8], [10], [11], [18], [22], [23], we might expect that a combination of age and contact structure would capture the observed data best (Figures 2D and 2G).

To assess how contributions from age and social contact behaviour influenced risk of infection, we compared model outputs with observed patterns of infection in each group. First, we used a simulation study to test whether our model could correctly identify the ‘true’ model among a range of candidate models. We simulated data for each of the 762 participants from a model with a specific number of age and contact classes and contact type (see Supplementary Text S1 for details), then compared model performance by considering the difference in Akaike Information Criterion [25] (ΔAIC) for each possible model in our framework. For four different simulation models, our framework correctly identified the structure of the original model each time (Figure S1).

**Figure 3:**
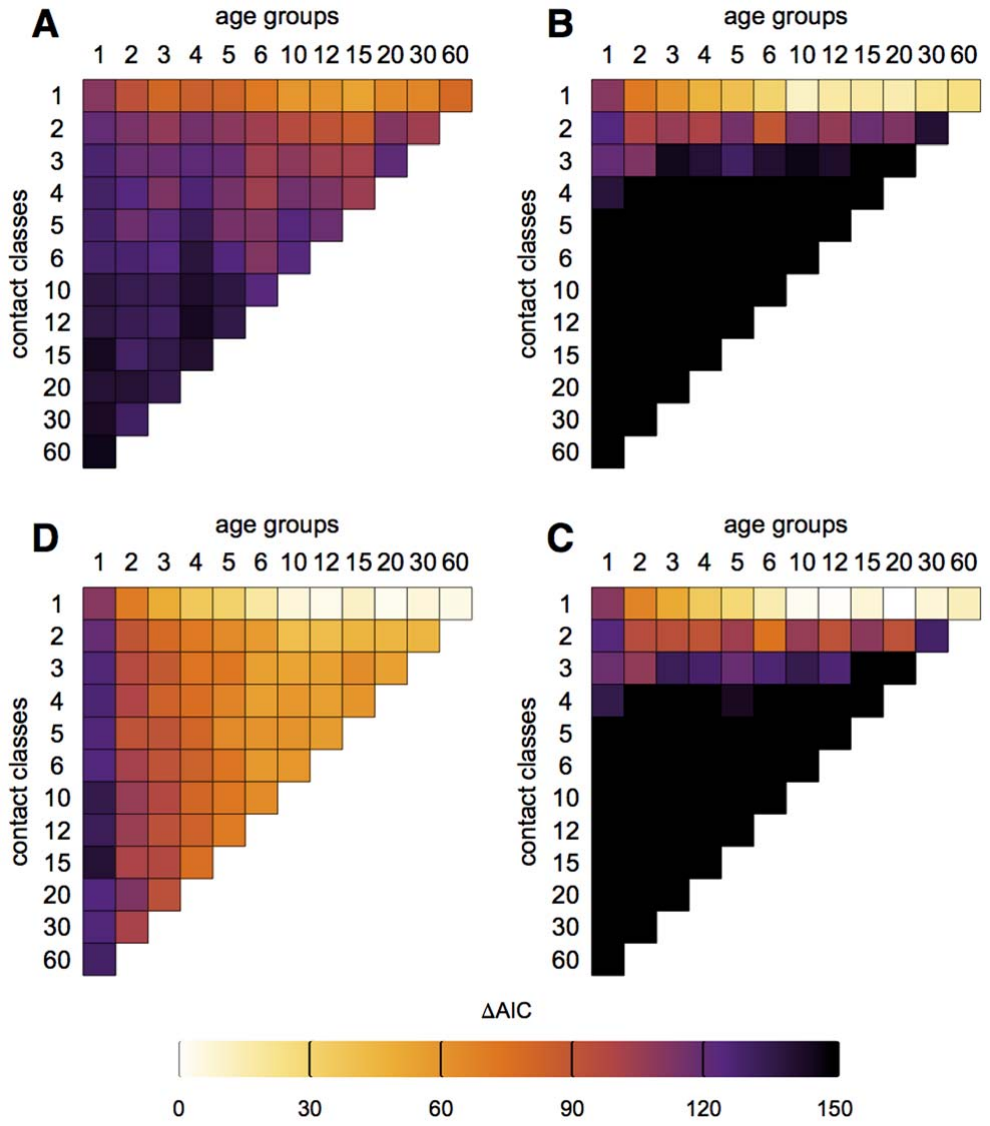
Comparison of different models in Figure 1A. (A) Model based on all contacts with relative susceptibility of over-18s, *α*, equal to one. (B) Model based on close contacts with *α* = 1. (C) Model based on all contacts with variable *α*. (D) Model based on close contacts with variable *α*. Colour shows model support under the Akaike Information Criterion (AIC). Note that models with numerous contact classes in B and D had some classes consisting solely of individuals – some of whom had been infected – that had no reported close contacts. The likelihood of such people seeing infection given the model assumptions was zero; the difference in AIC was therefore infinite.

Having tested the robustness of our inference method, we considered infection data from the 2009 pandemic in Hong Kong. Figures 3A–B show the performance of models with different numbers of age groups and contact classes. We found that age-based models, parameterised by the average social behaviour of each age group, gave the most parsimonious explanation of our data. The best performing model according to the difference in Akaike Information Criterion had 10 age groups, with only one contact class in each, and assumed transmission was driven by reported close contacts. In both Figures 3A and 3B, additional contact classes led to worse model performance: the best performing models assumed homogeneous mixing within each age group.

Models incorporating transmission based on close contacts gave a good prediction when there were few contact classes, but a very poor prediction when within-age group contact resolution increased (Figure 3B). Some of the models with multiple contact classes in Figure 3B had classes consisting solely of individuals – some of whom had been infected – that had no reported close contacts. The likelihood of such people seeing infection given the model assumptions was therefore zero. To assess whether our results were sensitive to these assumptions, we considered a framework with an additional small background rate of random contact among all members of the population (see Supplementary Text S1 for details). This extra parameter resulted in a non-zero likelihood for all age all contact classes (Figure S2), but did not change the overall pattern in Figures 3A–B.

The best performing model in Figure 3B underestimated attack rates in the under 18s (Figure S3). This was likely because we had not accounted for differences in susceptibility between older and younger age groups to the influenza A/H1N1p virus [26]. Therefore we also considered a model in which the relative degree of susceptibility of over-18s could vary (details in Text S1), denoted by parameter *α*. With the addition of *α* to the basic reproduction number, *R_0_*, we were using only two free parameters. Figures 3C–D show the performance of different models when this additional parameter was included. The qualitative pattern remained the same, but there was a significant reduction in the AIC for many of the models. The best-supported model, which had 20 age groups, was not significantly different than the saturated model for 20 age groups (likelihood ratio test, 18 degrees of freedom, p-value = 0.993). Even considering the multiple model comparisons in our study, the similarity between the saturated likelihood and our best fit two-parameter likelihood suggests that this framework effectively captures key aspects of these data.

**Figure 4:**
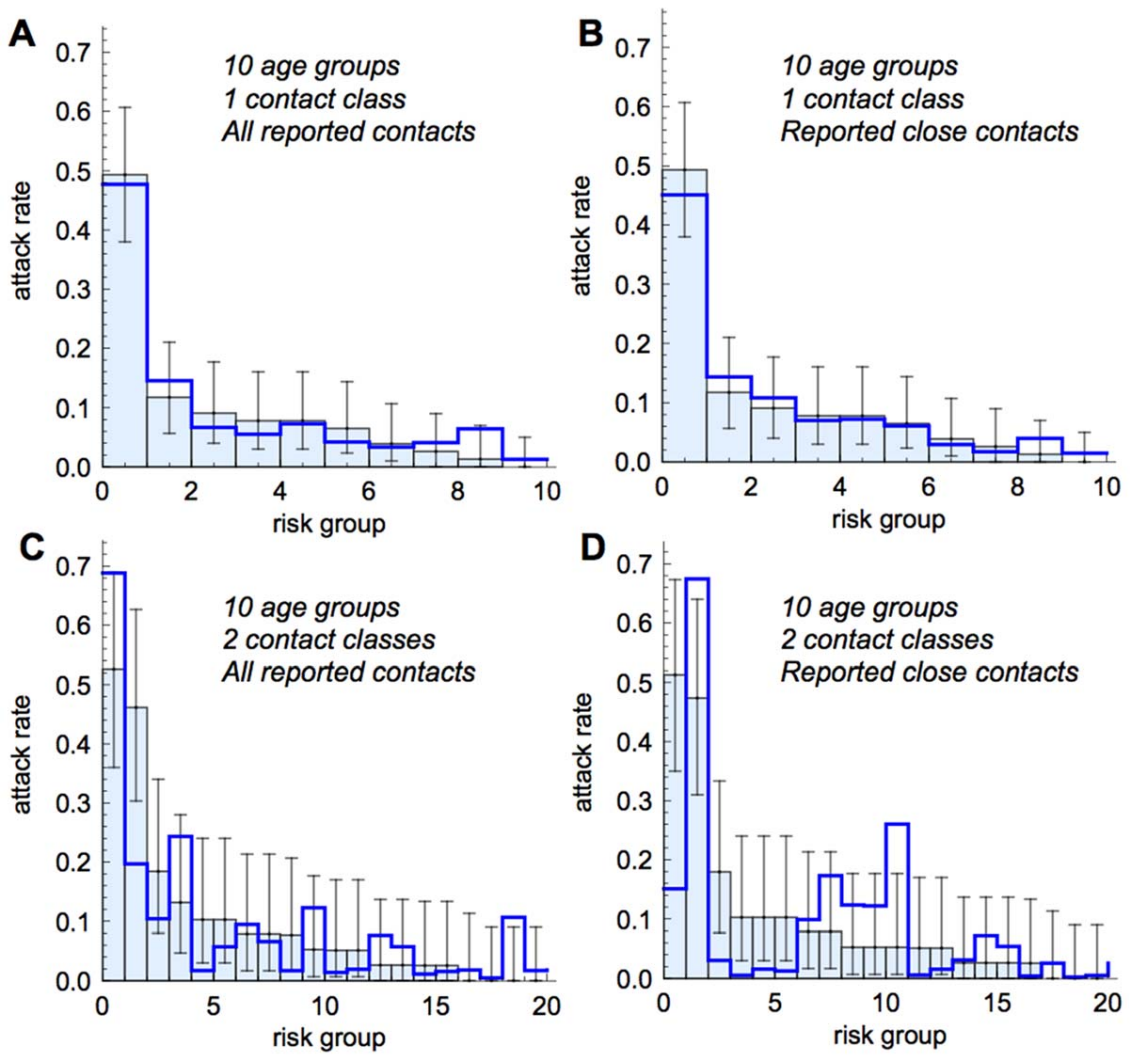
Comparison of model fits to data, with classes sorted by empirically observed risk of infection. Thick blue line, model prediction; light blue bars, data. Error bars give 95% binomial confidence interval. (A) Model based on all contacts with 10 age groups and 1 contact class in each. (B) Model based on close contacts with 10 age groups and 1 contact class in each. (C) Model based on all contacts with 10 age groups and 2 contact classes. (D) Model based on close contacts with 10 age groups and 2 contact classes in each. All models have variable relative susceptibility in the over-18s.

By examining the difference between observed and predicted values we were able to illustrate the reason for the decrease in AIC with increased contact classes. When the population is divided into 10 age groups, and these groups are sorted by the observed attack rate in each, the output from models using all reported contacts (Figure 4A) and close contacts (Figure 4B) is consistent with real patterns of infection. The addition of a second contact class in each age group, creating a total of 20 risk groups, leads to substantially worse performance, with models failing to capture observed attack rates in most at-risk groups by a substantial margin (Figures 4C–D). However, it is interesting that when all reported contacts are incorporated into a model with two contact classes per age group (Figure 4C), model predictions are closer to the observed data than when close contacts are used (Figure 4D).

**Figure 5:**
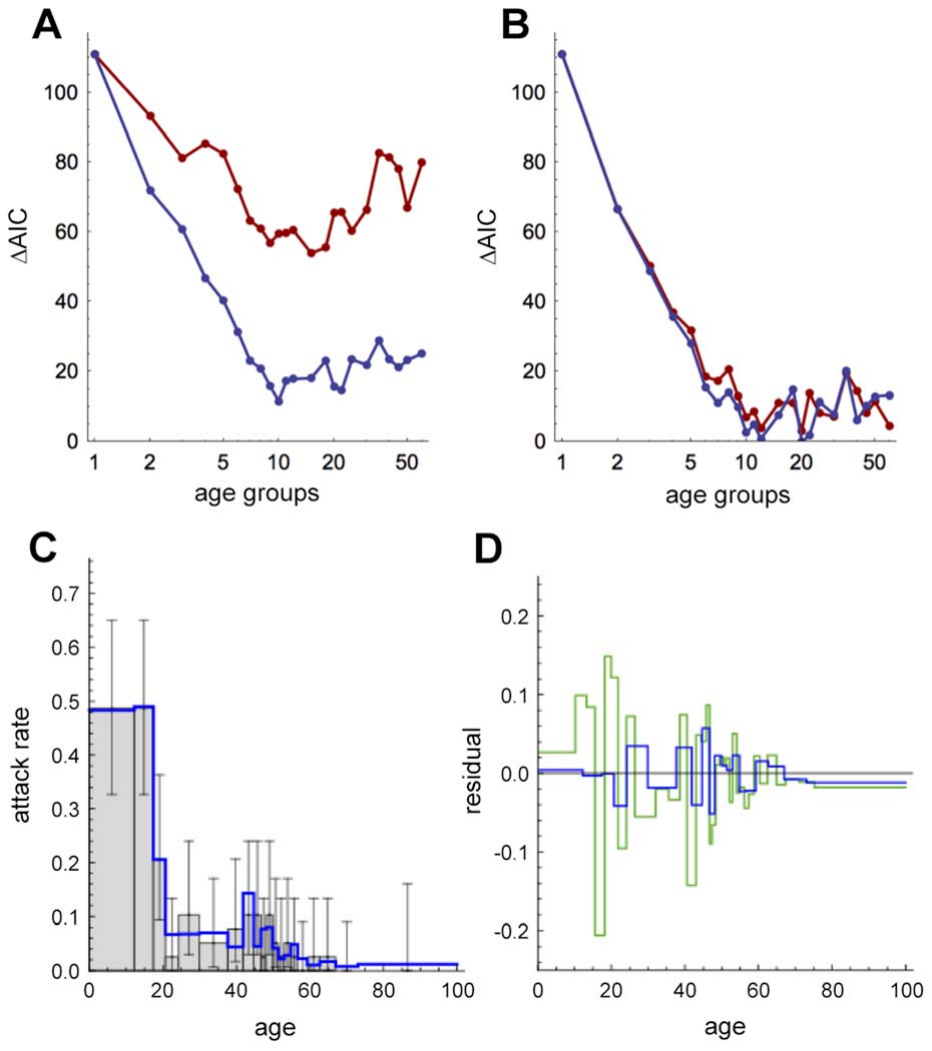
The social resolution of influenza transmission. (A) Detailed analysis of AIC for models with age structure only and *α* = 0 (i.e. top rows in Figure 3A–B), with transmission based on: red, total contacts; blue, close contacts. (B) AIC for age-structured models with variable *α*. (C) Performance of best-supported model in Figure 4B, which has 20 age groups and transmission based on close contacts, against data. Light grey bars show observed proportion of individuals that are seropositive, with 95% binomial confidence interval given by error bars. Blue solid line shows model prediction. (D) Comparison of residuals for model in Figure 4C (blue line) and equivalent model with 35 age groups (green line).

Figures 5A–5B shows the performance of the age-only models (i.e. the top row in each grid in Figures 3A–D) as the number of age groups increased in small increments. When all ages were equally susceptible, models using close contacts performed significantly better than their counterparts based on total contacts (Figure 5A). When we allowed relative susceptibility in the over 18s to vary, models incorporating close and total contacts both had similar levels of support (Figure 5B), although the model with transmission based on total contacts required a much lower relative susceptibility in older ages (Figure S4). Estimates for the basic reproduction number, *R_0_*, are shown in Table S1. As before, the best performing model included transmission based on close contacts (Tables S2). Overall, the results were robust to choice of age cut-off for relative susceptibility: having reduced susceptibility in the over-10s or over-30s instead of over-18s did not substantially change the overall pattern of the AIC (Figure S5).

We used two types of data in our analysis: the reported social contact data and the paired sera. Although we accounted for the observation error in the sera with the Bernoulli distributed likelihood terms in Equation 2, our framework made the assumption that social contacts in our sample were representative of the population. To test the sensitivity of results in Figure 5B to this assumption, we repeated our analysis using alternative datasets generated using bootstrap samples of the Hong Kong data (details in Text S1). The pattern of improvement in model performance as the number of age groups increased remained consistent when these bootstrap datasets were used (Figure S6).

We also assessed how our mechanistic model, which predicted final epidemic size (Equation 1), compared with a simple statistical framework. We considered a two-parameter logistic regression model that predicted infection risk using reported contacts only. Although the regression model performed well when there are 10 age groups, performance grew significantly worse as the resolution of the model increased (Figure S7). In contrast, the final size model generally continued to perform well once a high enough resolution was reached (Figure 5A–B), suggesting that the well-supported regression model relied on the age group boundaries falling at specific intervals (Figure S5), which happened to occur when there were 10 age groups (Figure S8). There was a fundamental difference in the structure of these two models: only the final-size model accounted for infection from secondary and tertiary contacts.

Figure 5C shows age-specific risk of infection in the overall best performing model, which had 20 age groups and variable susceptibility in the over-18s. The model reproduced the observed drop in risk of infection after childhood, and the small peak that occurs in individuals of parental age. In contrast, the equivalent model with 35 age classes did not reproduce this pattern as well (Figure 5D), and hence had less support.

Although we did not use data on which participants were parents, the rise in observed infection risk correlates well with the age groups that reported having a child in their household (Figure S9). To assess which component of the adults' contacts was driving the rise in infection risk (the within-group contacts to other adults or the between-group contacts to other ages), we assumed that certain age groups had no reported contacts with individuals aged 35–50. We saw little change in model performance if we removed the contacts – and hence contribution to the force of infection – of age groups over 20 (Table S3). However, there was a substantial reduction in performance if we assumed that individuals under 20 reported no contacts in the 35–50 age group, and hence made no contribution to force of infection acting upon the 35–50 group. These results suggest that children, through interactions with their peers and their parents, were responsible for much of the observed infection patterns.

## Discussion

Using a flexible model framework in which the population was structured by age and/or self-reported contacts, we compared theoretical predictions with serologically confirmed infection taken from a study of influenza A/H1N1p in Hong Kong [24]. We found strong evidence that an individual's risk of infection was influenced by the average social mixing behaviour of their age group, rather than by their personal reported contacts. Further, we found that finely resolved age classes were required to reproduce the observed distribution of infection. Our results suggest that the post-childhood drop and subsequent parental rise in social mixing are a crucial component of the transmission dynamics of respiratory pathogens like influenza.

There are some limitations to our study. We have only considered contact and serological data from Hong Kong: it would be helpful to test transmission models against observed disease prevalence in other countries. Moreover, we assumed that the one-day contact survey was representative of an individual's behaviour over the period of the epidemic. It would be interesting to see to what extent individual contact patterns vary over time: such changes could be measured in a longitudinal study, and compared with population-level variance in number of contacts.

We also used a single parameter to control the relative susceptibility of individuals who were over a specified age. However, a more detailed parameterisation may be required for other viruses, such as seasonal influenza strains [27]. Finally, although participants provided information on their number of contacts with each age group, we did not know which contact class these reported contacts were in. It was therefore necessary to infer interactions between different contact classes from the original egocentric data (see Supplementary Text S1). The age distribution of contacts of individuals in low, medium and high-contact groups follows a similar pattern (Figure S10), which suggests that this assumption of independence is reasonable, at least when it comes to modelling the age-specific of force of infection between different contact classes. However, factors such as clustering and location may also have an effect on the distribution of contacts: a future challenge would be to develop techniques that could incorporate such information and examine the impact on dynamics.

Social contact data can also be collected using electronic proximity sensors, which automatically record participants' interactions, rather than diary-based questionnaires. Such approaches can provide high-resolution information about the frequency and structure of contacts between participants [28], [29]. However, in a large community, a questionnaire-based approach has the advantage that contacts are recorded regardless of whether they wore sensors or not: it is not necessary to include all potential contacts in the study. Therefore, we suggest that both methods have merit, but that self-reported diary-based methods are perhaps the most useful currently because they can be applied to much larger study populations than device-based methods.

The results we present here build directly on recent statistical analysis of these same data in which it was concluded that, at the individual-level, a participant's self-reported social contacts alone were not a good predictor of their odds of influenza infection [30]. Specifically, an explicit age term was always also required when alternate explanatory variables were compared. These two sets of analyses are not inconsistent. The final size model presented here captures the combined risk of multiple generations of infection in the age-specific mixing matrix. Also, in the empirical study itself, we were not able to ask about behaviour immediately prior to infection. If we were to use a case-control design with confirmed currently infectious individuals as cases, we may find a much better correspondence between self-reported contacts and individual infection.

Of the models we tested, the best performing model included transmission based on close contacts. Previous work also has suggested that reported close contacts are a better proxy for parvovirus [11], varicella [11] and influenza [27] transmission than total contacts. However, it is still not clear which types of contact lead to transmission of influenza and how (or if) these risky contacts are reported in surveys of social contacts. Further, models with a large number of age groups generally perform worse under the AIC than models with 10–25 groups (Figures 5A–B). This might be owing to the sample size we used: at a fine resolution, mixing patterns are informed by only a small group of individuals. Ideally, future studies would test transmission models against observed disease prevalence using larger cohorts.

Our results suggest that infection risk is strongly influenced by the average social mixing behaviour of a person's age group, rather than by their individual reported contacts. This demonstrates that self-reported contacts have useful epidemiological value, as the average behaviour of a population can be used to predict individual infection patterns. Further, we have identified the likely social resolution of influenza transmission during the 2009 Hong Kong pandemic. Although different countries have different social and demographic structure, if the key age transitions – specifically, the post-childhood drop in risky contacts and subsequent parental rise – are fundamentally important to epidemic dynamics, it should be possible to profile different countries' social structure and use these aggregate population data to tailor predictions about infection attack rates. Such information would be relatively straightforward to collect, and could prove valuable in the future when targeting potentially costly interventions during an outbreak.

## Materials and Methods

### Ethics statement

All study protocols and instruments were approved by the institutional review boards of the University of Hong Kong. Written informed consent was sought from each participant aged 18 or above. Written proxy consent was sought from the parent or guardian of all participants aged 17 or below. In addition, the written assent to participate was asked from participants of aged 7 or above and 17 or below.

### Data

We used age and contact data similar to that in the POLYMOD study [6], but taken from a 2009/10 survey of 762 participants in Hong Kong [24]. Participants were recruited by random calling of residential landline numbers for Hong Kong. Data were collected by embedding an interviewer-led social contact questionnaire within a serological survey of influenza. On an assigned day, participants recorded contacts who they touched or had a face-to-face conversation with. The mean number of total contacts reported across all participants was 17.5; the median was 8.0. The frequency distribution of contact had a long tail, and we did not find evidence of a preference for reporting ‘round’ numbers ending in ‘0’ or ‘5’ (Figure S11).

As well as a social contact survey, paired sera were used to identify which of the participants had been infected. This was defined as a four-fold or more rise in titre, as measured by a neutralization assay, between baseline and follow-up visit. The assay tested for neutralizing antibody against influenza A/H1N1p. Such tests have been shown to be more sensitive than hemagglutination-inhibition assays: in a 2010 study, also conducted in Hong Kong, 18 of 19 individuals with virologically confirmed A/H1N1p infection exhibited at least a four-fold rise in neutralization titre [31].

Baseline samples were taken between 4 July 2009 and 19 September 2009. Once clinical surveillance indicated that the peak level of transmission had passed, follow-up samples were obtained between 11 November 2009 and 6 February 2010.

In the Hong Kong serological survey, participants could report contacts as being in one of three age groups: age under 20, 20 to 65, over 65. Relative to population size for each age group, under 20s reported fewer contacts with older groups than older groups reported with under 20s. We therefore adjusted the reported values to ensure reciprocity in contacts between each pair of age groups: if *m_a,b_* was the mean number of contacts in group a reported by individuals in group *b* and *P_a_* was the proportion of the population in age group *a*, we used a maximum likelihood approach [8] to obtain estimates that satisfied *m_a,b_P_b_* = *m_b,a_P_a_*.

### Model

We constructed a flexible model framework with which to compare different mechanistic explanations for infection risk, under the assumption that both age and contact behaviour were known. To construct a model with *A* age groups and *C* contact classes, we first sorted participants by age and divided them into *A* groups, each containing an equal number of people; the final class contained fewer individuals if there was a remainder after division. We then divided each age group into a further *C* classes, based on reported contacts. The contact classes for each age group were defined by sorting the individual reported number of contacts into ascending order, then dividing the age group into *C* equal parts. The output from each model was the final epidemic size, defined as the proportion of individuals infected in each age and contact group. As a result, we had only one model parameter to specify: the basic reproduction number, *R_0_*. In this section, we outline the general model framework, which could be used with any set of reported social contact data; the technical details of how the 2009 Hong Kong dataset was incorporated into the framework are given in Supplementary Text S1.

We used an SIR model for simulations, with individuals falling into one of three compartments: susceptible, infective or recovered (and hence immune). The force of infection acting on age-contact class *(a,i)* as a result of infectives in age-contact class *(b,j)* was proportional to two things: the mean number of contacts made by members of *(b,j)* with age group *a*, and the fraction of total contacts made by age group a that were with individuals in class *(a,i)*. We defined *m_ai,bj_* as the mean number of contacts with individuals in age group a and contact class *i* by participants in age group *b* and class *j*. The transmission rate to group (*a,i*) from group (*b,j*) was therefore given by *β_ai,bj_* = *qm_ai,bj_*/*P_ai_*, where *q* was a scaling factor dependent on the basic reproduction number and *P_ai_* was the proportion of the population in group (*a,i*) [8]. The final epidemic size in each age-contact class (*a,i*), *φ_ai_*, could therefore be found by solving the following coupled equation [32],
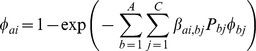



In our framework, a population could be divided into arbitrarily finely resolved age and contact classes (although the maximum number of possible classes – and hence model resolution – would ultimately be constrained by the total number of participants in the social contact survey). Most modelling studies incorporating age-stratified social contact data used between six and twenty age groups [8]–[20]. In contrast, other studies have explored the effects of the distribution of number of contacts on final epidemic size [7], [22], [23], without using age-structure. Our framework encompassed all of these assumptions: depending on how many age groups and contact classes included, the framework produced a simple mass-action model, an age-structured formulation, or a model dependent only on the degree distribution of contacts (Figure 1). As there were only 762 participants in the Hong Kong study, we limited the maximum possible number of age and/or contact groups in a model to 60, to avoid groups containing too few people (Figure 2A).

### Relatively susceptibility in older groups

There was evidence that older age groups had some pre-existing immunity to the 2009 influenza A/H1N1p strain [2], [26]. We included an additional parameter to reflect this immunity: individuals above a certain age had their susceptibility reduced by a factor α, where 0<*α*≤1. The cut-off could vary: in our analysis we considered a reduction in over-10s, over-18s and over-30s (details in Supplementary Text S1).

### Statistical inference

Given a set of parameters, *θ*, we denoted the model prediction for attack rate in age group *a* and contact class *i* by *φ_ai_(θ)*. Let *Y* denote the set of neutralization titres for the study group, and *Y_k_* denote the titres for individual *k*. If individual *k* was aged *a* and in contact class *i*, the likelihood of *θ* given the data could therefore be calculated with a Bernouilli probability mass function,
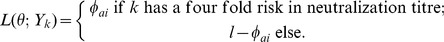



We then found the parameter set *θ* that maximises the log-likelihood across all individuals,
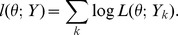



The models were compared using the Akaike Information Criterion (AIC) [25]. If a model contains *k* parameters then AIC = *2k–2l*. Here *k* = 1 in the basic transmission model, and *k* = 2 in the model with variable susceptibility. Note that with 762 participants and only one or two parameters, it makes negligible difference to our results whether we use AIC or AIC_c_, the criterion corrected for low sample size. For each model, we calculate ΔAIC, the difference between the AIC for that model and the AIC of the model with the lowest AIC. The following approximate rules have been suggested when using this measure [25]: models with ΔAIC≤2 have substantial support compared to the best model; those with 4≤ΔAIC≤7 have much less; those with ΔAIC>10 have practically no support compared with the best model given the data.

## Supporting Information

Figure S1 **Identification of true model using simulated data.** First we simulated data for each of the 762 participants from a model with a specific number of age and contact classes and contact type (see Supplementary Text S1 for details). We then calculated model support under the Akaike Information Criterion for each possible model in our framework. The left column shows AIC for models based on all contacts; the right column shows results from models using close contacts. Each row uses simulated data from one of four different ‘true’ models. (A) and (B), data simulated using model with 10 age groups and 1 contact class, and all reported contacts. The correct model is indicated with a blue ‘X’. (C) and (D), data simulated using model with 10 age groups and 1 contact class, and reported close contacts. (E) and (F), data simulated using model with 1 age group and 10 contact classes, and all reported contacts. (G) and (H), data simulated using model with 10 age groups and 1 contact class, and reported close contacts.
(TIFF)

Figure S2 **Similar plots to** **Figure 1A and B** **with a small background risk of infection included.** There are 10 age groups, with only one contact class in each, with transmission based on reported close contacts.
(TIFF)

Figure S3 **Risk of infection in best model of those shown in** **Figures 2A–B** **.** There are 10 age groups, with only one contact class in each, with transmission based on reported close contacts.
(TIFF)

Figure S4 **Maximum likelihood point estimate for relative susceptibility of over 18s,** ***α*** **, as number of age groups varies.** Red line shows model using total reported contacts; blue, model using close contacts.
(TIFF)

Figure S5 **Sensitivity of results to different cut offs for drop in susceptibility.** (A) Analysis of ΔAIC for models with age structure only and variable *α* for age groups above 10, with transmission based on: red, total contacts; blue, close contacts. (B) ΔAIC for models with variable *α* for age groups above 30.
(TIFF)

Figure S6 **Sensitivity of results in** **Figure 5B** **to different social contact data.** (A) ΔAIC for age-structured models with variable *α*, with transmission based on total reported contacts. Each line represents results from inference performed using a bootstrap resample of the Hong Kong data. Ten such samples were performed: each is shown in a different colour. (B) ΔAIC for age-structured models with variable *α*, with transmission based on total reported contacts.
(TIFF)

Figure S7
**ΔAIC for logistic regression model as number of age groups varies.** Transmission is based on: red, total contacts; blue, close contacts. The two parameter logistic regression model predicts risk from reported contacts only. For contact class i within age group a, risk of infection is given by *φ_ai_* = 1/(exp [−(*μ_0_+μ_1_M_ai_*)]+1) where 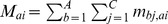 and *μ_0_* and *μ_1_* are parameters to be fitted.(TIFF)

Figure S8 **Age boundaries used for different numbers of age groups.** Groups are defined by sorting the 762 survey participants by age and dividing them into A groups, each containing an equal number of people.
(TIFF)

Figure S9 **Proportion of each age group in** **Figure 4C** **that reported having a child in their household.**
(TIFF)

Figure S10 **Age distribution of contacts made with different contact classes in model.** We constructed a model with 10 age groups, each containing 3 contact classes. For each age group, we plotted age distribution of contacts made with age of the three contact classes. Red points, low-contact class (the third of the age group with fewest reported contacts); green points, middle contact class; blue points, high-contact class (third of age group with most reported contacts). Age boundaries for the 10 age groups are shown in Figure S8.
(TIFF)

Figure S11 **Frequency distribution of contacts.** Distribution of total reported contacts across all 762 study participants. Numbers that end in ‘0’ or ‘5’ are indicated by red points: these do not appear to be reported more frequently than neighbouring numbers.
(TIFF)

Table S1 **Maximum likelihood point estimate for** ***R_0_*** **in different models, arranged by contacts used.**
(PDF)

Table S2 **Difference in AIC between the best performing model (in bold) and other models, arranged by contacts used.**
(PDF)

Table S3 **Change in model performance when different components of the force of infection into groups aged 35–50 are omitted.**
(PDF)

Text S1 **The contribution of social behaviour to the transmission of influenza A in a human population.**
(PDF)
