# Supplementary figures and images for: The Contribution of Social Behaviour to the Transmission of Influenza A in a Human Population
Source: PLoS Pathog. 2014 Jun 26;10(6):e1004206. doi: 10.1371/journal.ppat.1004206 (PMC4072802; doi:10.1371/journal.ppat.1004206)

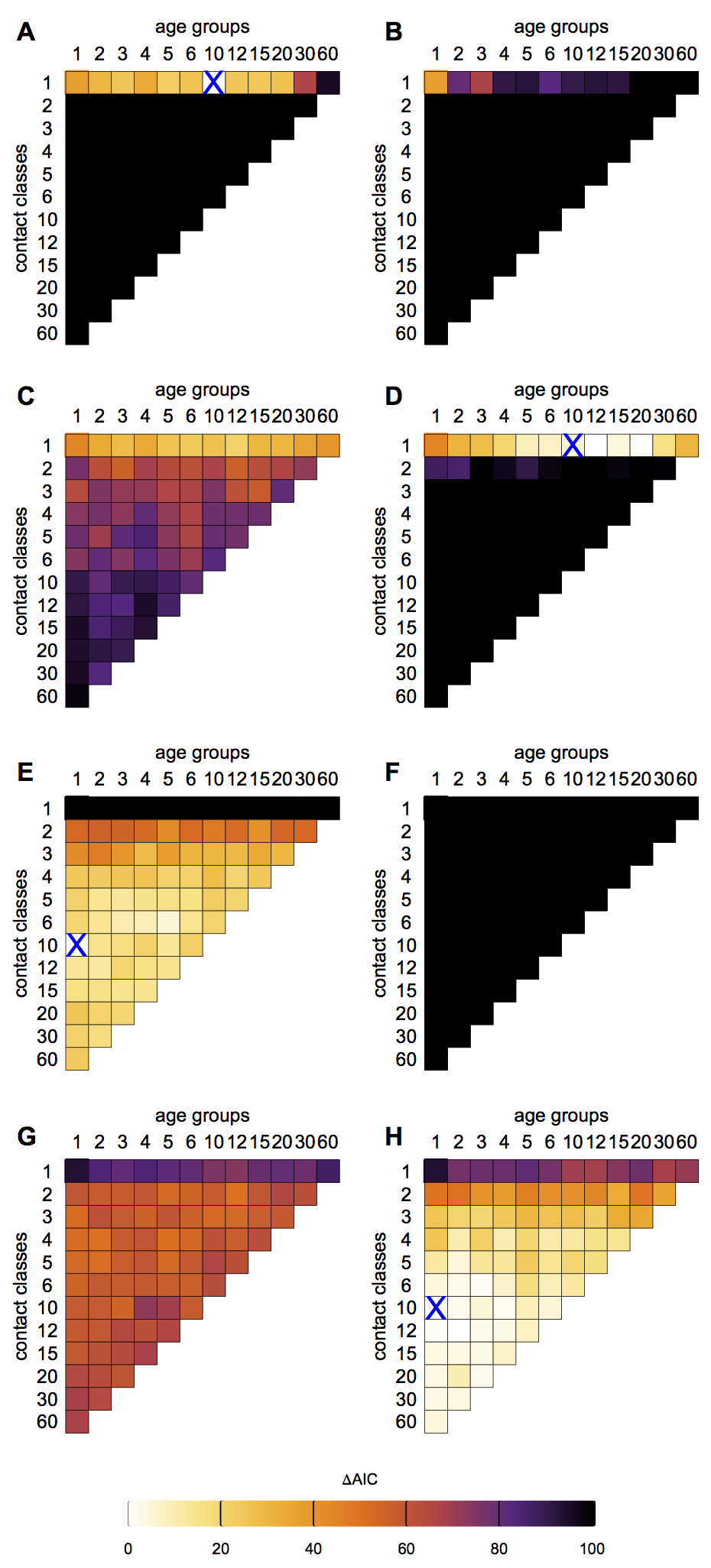

Supplement: Figure S1 — Identification of true model using simulated data. First we simulated data for each of the 762 participants from a model with a specific number of age and contact classes and contact type (see Supplementary Text S1 for details). We then calculated model support under the Akaike Information Criterion for each possible model in our framework. The left column shows AIC for models based on all contacts; the right column shows results from models using close contacts. Each row uses simulated data from one of four different ‘true’ models. (A) and (B), data simulated using model with 10 age groups and 1 contact class, and all reported contacts. The correct model is indicated with a blue ‘X’. (C) and (D), data simulated using model with 10 age groups and 1 contact class, and reported close contacts. (E) and (F), data simulated using model with 1 age group and 10 contact classes, and all reported contacts. (G) and (H), data simulated using model with 10 age groups and 1 contact class, and reported close contacts. (TIFF) [file ppat.1004206.s001.tiff]

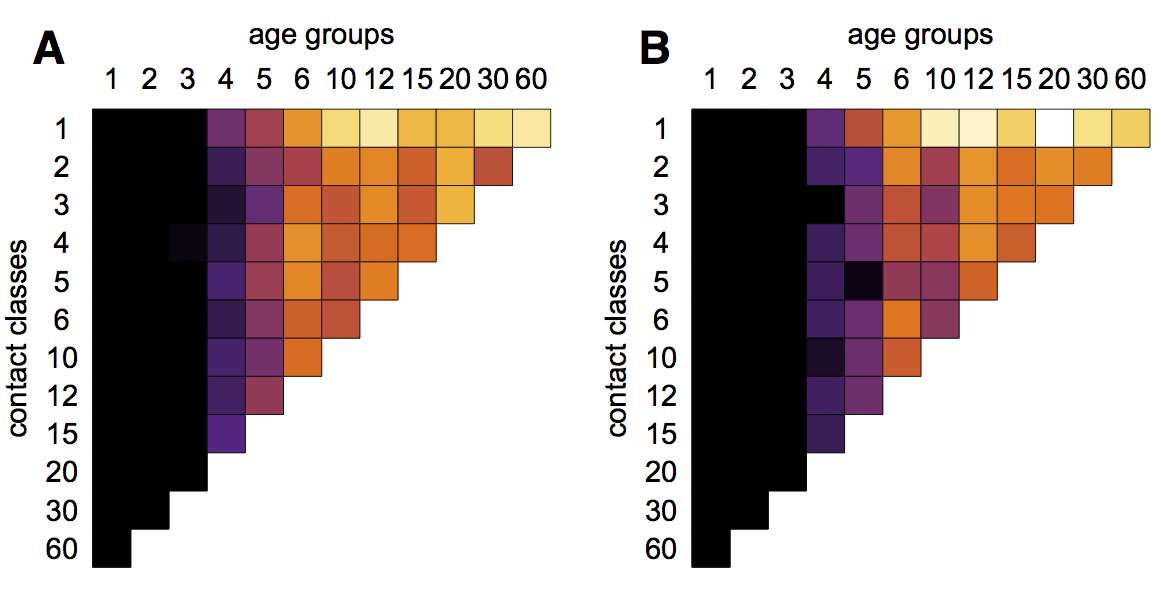

Supplement: Figure S2 — Similar plots to Figure 1A and B with a small background risk of infection included. There are 10 age groups, with only one contact class in each, with transmission based on reported close contacts. (TIFF) [file ppat.1004206.s002.tiff]

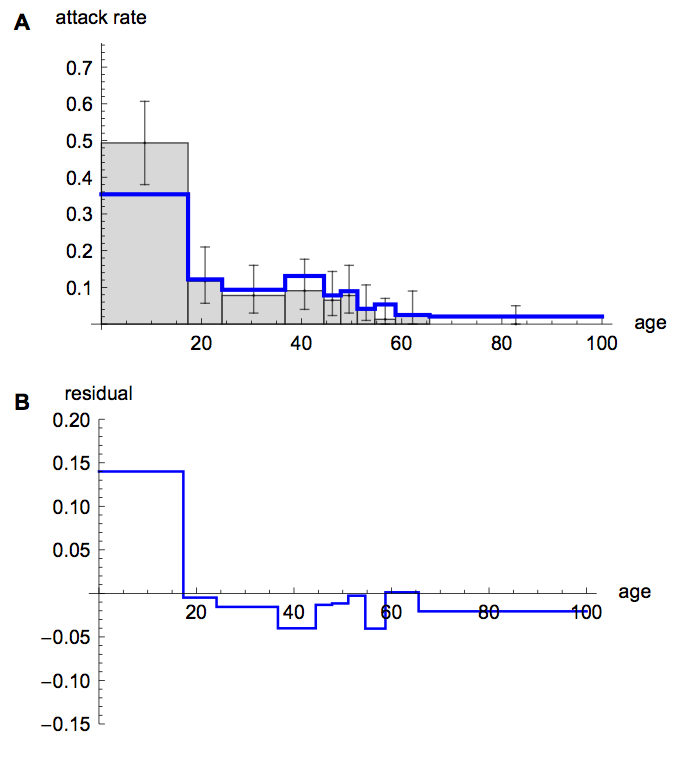

Supplement: Figure S3 — Risk of infection in best model of those shown in Figures 2A–B . There are 10 age groups, with only one contact class in each, with transmission based on reported close contacts. (TIFF) [file ppat.1004206.s003.tiff]

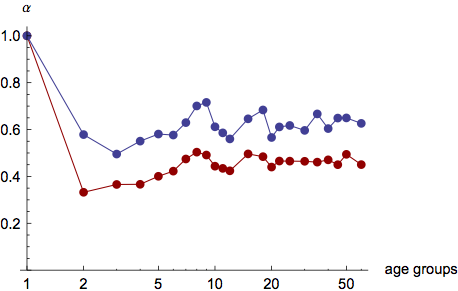

Supplement: Figure S4 — Maximum likelihood point estimate for relative susceptibility of over 18s, α , as number of age groups varies. Red line shows model using total reported contacts; blue, model using close contacts. (TIFF) [file ppat.1004206.s004.tiff]

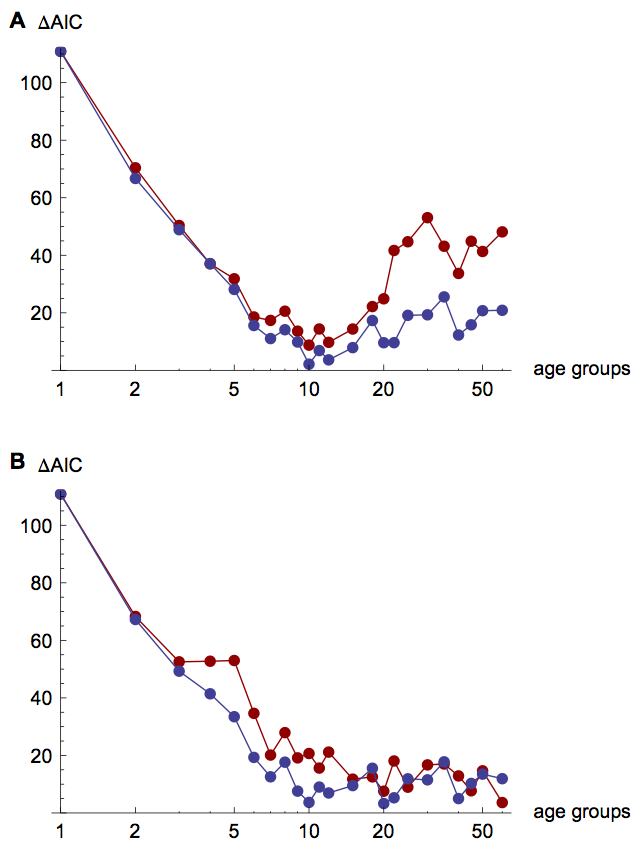

Supplement: Figure S5 — Sensitivity of results to different cut offs for drop in susceptibility. (A) Analysis of ΔAIC for models with age structure only and variable α for age groups above 10, with transmission based on: red, total contacts; blue, close contacts. (B) ΔAIC for models with variable α for age groups above 30. (TIFF) [file ppat.1004206.s005.tiff]

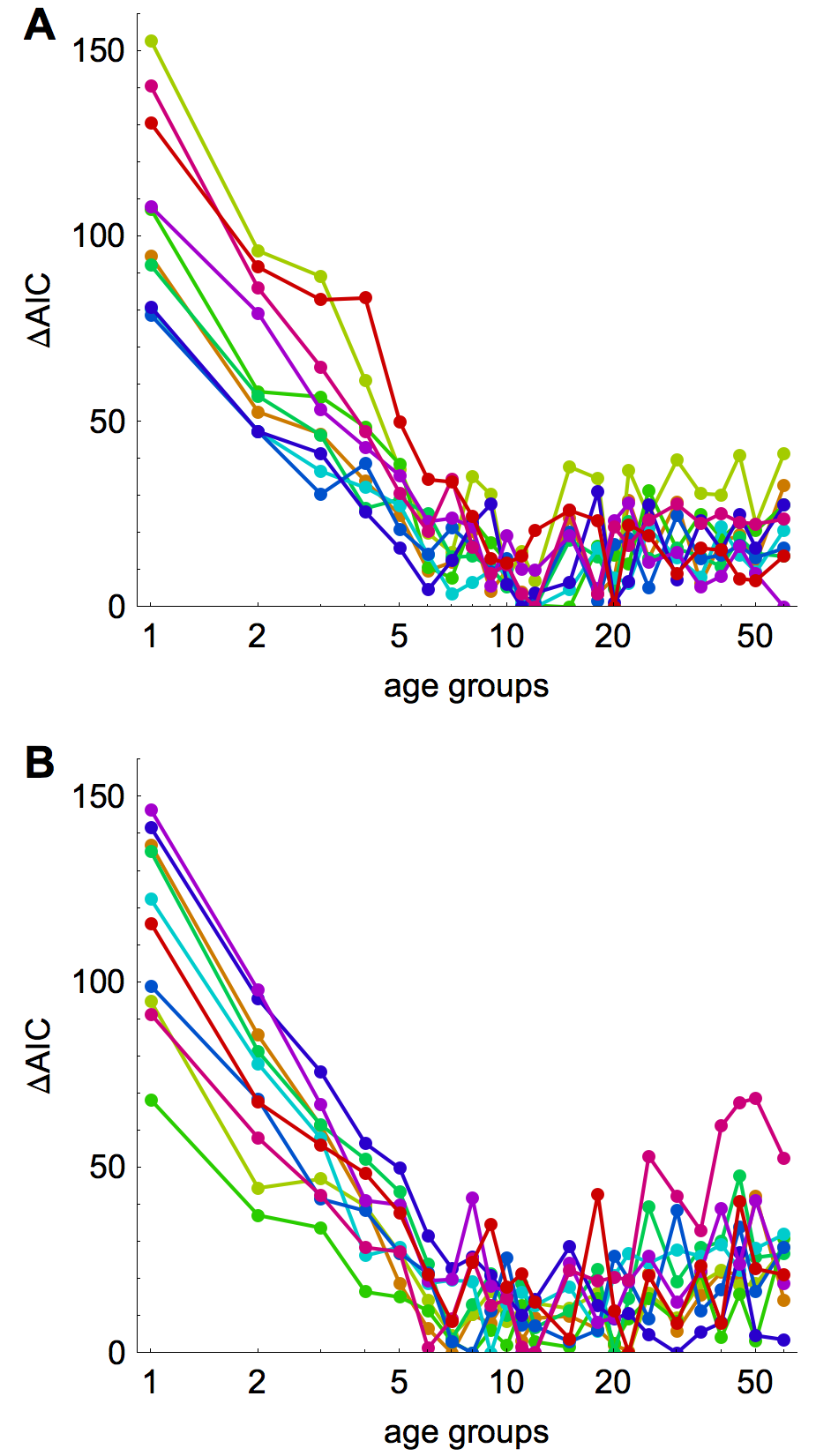

Supplement: Figure S6 — Sensitivity of results in Figure 5B to different social contact data. (A) ΔAIC for age-structured models with variable α, with transmission based on total reported contacts. Each line represents results from inference performed using a bootstrap resample of the Hong Kong data. Ten such samples were performed: each is shown in a different colour. (B) ΔAIC for age-structured models with variable α, with transmission based on total reported contacts. (TIFF) [file ppat.1004206.s006.tiff]

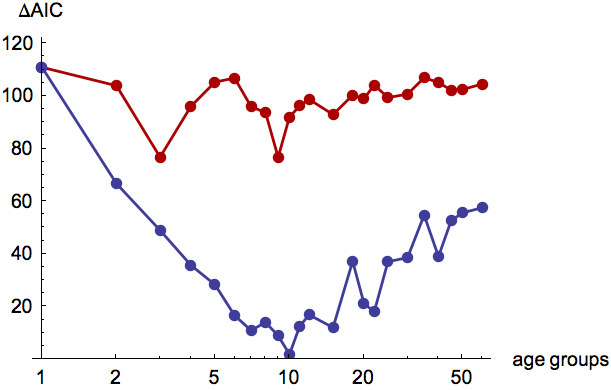

Supplement: Figure S7 — ΔAIC for logistic regression model as number of age groups varies. Transmission is based on: red, total contacts; blue, close contacts. The two parameter logistic regression model predicts risk from reported contacts only. For contact class i within age group a, risk of infection is given by φai = 1/(exp [−(μ0+μ1Mai)]+1) where and μ0 and μ1 are parameters to be fitted. (TIFF) [file ppat.1004206.s007.tiff]

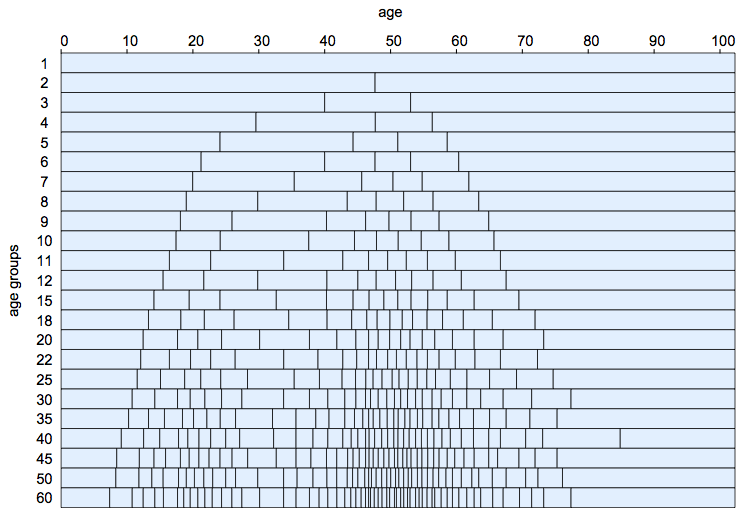

Supplement: Figure S8 — Age boundaries used for different numbers of age groups. Groups are defined by sorting the 762 survey participants by age and dividing them into A groups, each containing an equal number of people. (TIFF) [file ppat.1004206.s008.tiff]

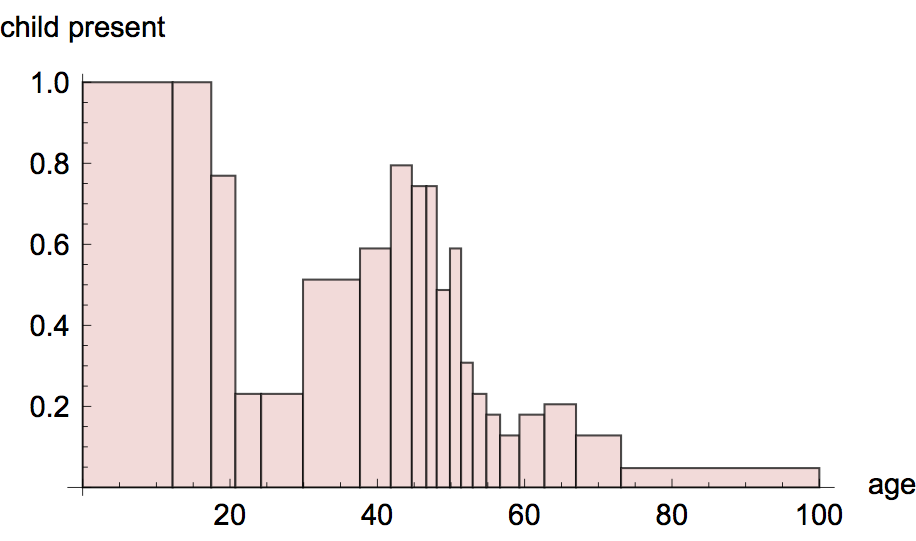

Supplement: Figure S9 — Proportion of each age group in Figure 4C that reported having a child in their household. (TIFF) [file ppat.1004206.s009.tiff]

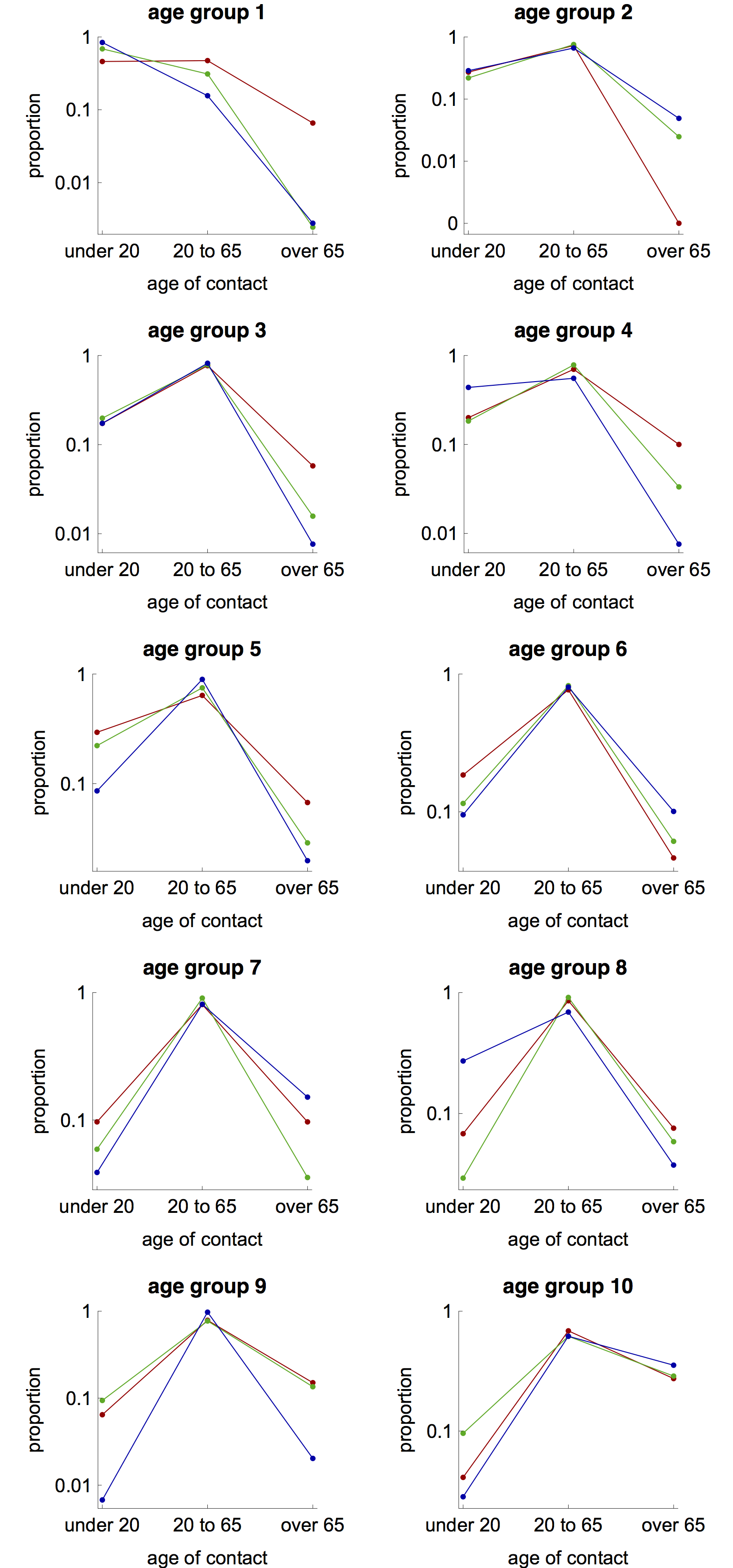

Supplement: Figure S10 — Age distribution of contacts made with different contact classes in model. We constructed a model with 10 age groups, each containing 3 contact classes. For each age group, we plotted age distribution of contacts made with age of the three contact classes. Red points, low-contact class (the third of the age group with fewest reported contacts); green points, middle contact class; blue points, high-contact class (third of age group with most reported contacts). Age boundaries for the 10 age groups are shown in Figure S8. (TIFF) [file ppat.1004206.s010.tiff]

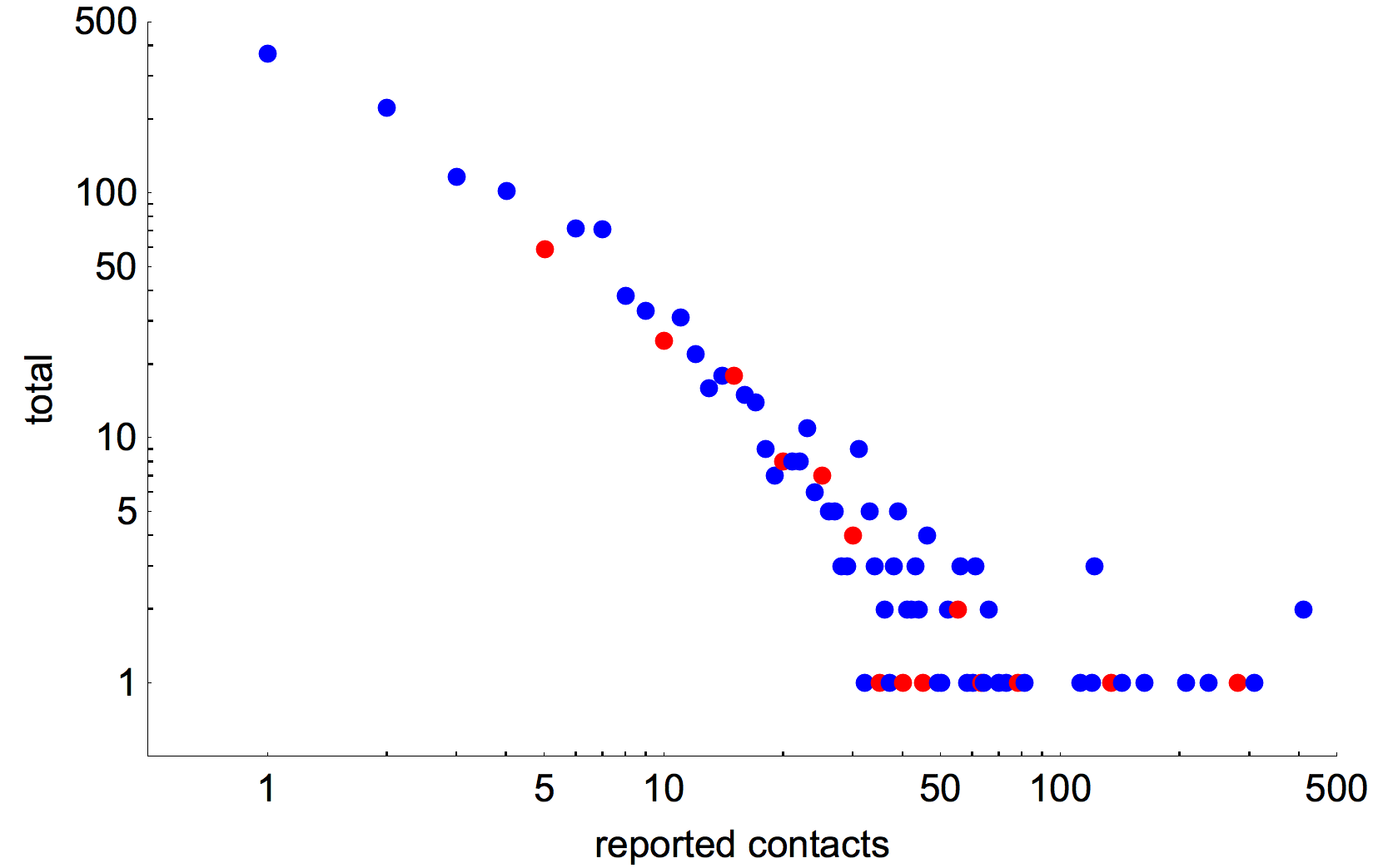

Supplement: Figure S11 — Frequency distribution of contacts. Distribution of total reported contacts across all 762 study participants. Numbers that end in ‘0’ or ‘5’ are indicated by red points: these do not appear to be reported more frequently than neighbouring numbers. (TIFF) [file ppat.1004206.s011.tiff]
