# Supplementary material for: The Contribution of Social Behaviour to the Transmission of Influenza A in a Human Population
Source: PLoS Pathog. 2014 Jun 26;10(6):e1004206. doi: 10.1371/journal.ppat.1004206 (PMC4072802; doi:10.1371/journal.ppat.1004206)
Supplement: Table S1 — Maximum likelihood point estimate for R0 in different models, arranged by contacts used. (PDF) [file ppat.1004206.s012.pdf]

Table S1. Maximum likelihood point estimate for R_0 in different models, arranged by contacts used.

Age groups	All (vary α)	Close (vary α)	All ($\alpha = 0$)	Close ($\alpha = 0$)
1	1.33	1.27	1.05	1.05
2	1.29	1.13	1.06	1.08
3	1.35	1.21	1.06	1.09
4	1.55	1.25	1.06	1.10
5	1.67	1.29	1.06	1.11
6	1.65	1.31	1.07	1.12
7	1.54	1.32	1.07	1.15
8	1.52	1.32	1.08	1.18
9	1.56	1.33	1.08	1.20
10	1.58	1.37	1.08	1.21
11	1.58	1.37	1.08	1.19
12	1.63	1.37	1.07	1.16
15	1.55	1.35	1.08	1.17
18	1.59	1.35	1.08	1.19
20	1.64	1.40	1.08	1.19
22	1.62	1.38	1.08	1.18
25	1.65	1.36	1.08	1.17
30	1.66	1.41	1.08	1.18
35	1.76	1.38	1.09	1.20
40	1.74	1.39	1.09	1.16
45	1.84	1.41	1.10	1.20
50	1.67	1.42	1.09	1.20
60	1.85	1.47	1.11	1.23
