# Supplementary material for: The Contribution of Social Behaviour to the Transmission of Influenza A in a Human Population
Source: PLoS Pathog. 2014 Jun 26;10(6):e1004206. doi: 10.1371/journal.ppat.1004206 (PMC4072802; doi:10.1371/journal.ppat.1004206)
Supplement: Table S2 — Difference in AIC between the best performing model (in bold) and other models, arranged by contacts used. (PDF) [file ppat.1004206.s013.pdf]

Table S2. Difference in AIC between the best performing model (in bold) and other models, arranged by contacts used.

Age groups	All (vary α)	Close (vary α)	All ($\alpha = 0$)	Close ($\alpha = 0$)
1	110.9	110.9	108.9	108.9
2	66.7	66.7	91.2	69.9
3	50.3	48.8	79.2	58.8
4	36.9	35.6	83.4	44.8
5	31.8	28.1	80.4	38.4
6	18.5	15.4	70.4	29.4
7	17.4	11.0	61.2	21.0
8	20.6	14.0	59.0	18.9
9	13.0	9.8	55.0	13.8
10	7.1	2.6	57.6	9.5
11	8.7	4.9	57.8	15.3
12	3.9	0.9	58.6	15.8
15	11.1	7.7	52.0	16.1
18	11.2	14.8	53.6	21.1
20	3.2	**0.0**	63.5	13.6
22	13.8	1.8	63.8	12.6
25	8.2	11.3	58.4	21.4
30	7.2	7.9	64.5	19.9
35	19.7	20.1	80.8	26.8
40	14.5	6.2	79.4	21.4
45	8.2	10.3	76.1	19.1
50	11.5	12.9	65.0	21.3
60	4.5	13.2	77.9	23.1
