# Supplementary material for: The Contribution of Social Behaviour to the Transmission of Influenza A in a Human Population
Source: PLoS Pathog. 2014 Jun 26;10(6):e1004206. doi: 10.1371/journal.ppat.1004206 (PMC4072802; doi:10.1371/journal.ppat.1004206)
Supplement: Table S3 — Change in model performance when different components of the force of infection into groups aged 35–50 are omitted. (PDF) [file ppat.1004206.s014.pdf]

Table S3. Change in model performance when different components of the force of infection into groups aged 35–50 are omitted.

Component omitted[1]	ΔAIC
none	0
under 20	8.8
20–34	0.6
35–50	0.4
over 50	0.1

[1] We assume that participants in the specified groups report no contacts with the 35–50 age groups (i.e. if age group b is omitted, and a is an age group in 35-50 range, $m_{a,b} = 0$). When no component of the force of infection is omitted, we obtain the best fitting model shown in Figure 5C. Each model is refitted to the data, and compared with alternatives using the AIC.
