# Supplementary material for: The Contribution of Social Behaviour to the Transmission of Influenza A in a Human Population
Source: PLoS Pathog. 2014 Jun 26;10(6):e1004206. doi: 10.1371/journal.ppat.1004206 (PMC4072802; doi:10.1371/journal.ppat.1004206)
Supplement: Text S1 — The contribution of social behaviour to the transmission of influenza A in a human population. (PDF) [file ppat.1004206.s015.pdf]

Text S1: The contribution of social behaviour to the transmission of influenza A in a human population

Model structure

To construct a model with A age groups and C contact classes, we first sorted participants by age and divided them into A groups, each containing an equal number of people; the final class contained fewer individuals if there was a remainder after division. We defined P_a to be the age distribution of these groups (for Hong Kong, these values were taken from the 2011 census [1]), where $\sum_{a=1}^{A} P_a = 1$. Using data on reported contacts we found $m_{a,b}$, the mean number of contacts in age group a reported by individuals in group b. We then divided each age group into a further C classes, based on reported contacts. The classes for each age group were defined by sorting the individual reported number of contacts into ascending order, then dividing the list into C equal parts (again, the final class was smaller if there was a remainder). If N_{ai} was the number of participants who were age a and in contact class i, the age distribution of that age and contact group was given by

$$P_{ai} = \frac{P_a N_{ai}}{\sum_{k=1}^{C} N_{ak}} \ . \tag{S1}$$

In social contact surveys, participants often report only the age group of whom they interacted with, and not their contact class [2, 3, 4]. We therefore had to estimate $m_{ai,bj}$, the mean number of contacts with individuals in age group a and contact class i by participants in age group b and class j. While we did not have data on the social contacts of reported contacts, we could account for the age distribution of contacts in different contact classes when constructing our transmission matrices. We knew how many of the contacts group (b, j) reported were in age group a, so only needed to estimate how these were split between the different contact classes in group a. We did this by assuming that the distribution of contacts made by (b, j) with group (a, i) followed a weighted average [5], based on the total contacts reported by all the contact classes in age group a:

$$m_{ai,bj} = m_{a,bj} \frac{m_{b,ai} P_{ai}}{\sum_{k=1}^{C} m_{b,ak} P_{ak}} \ . \tag{S2}$$

We used an SIR model for simulations, with individuals falling into one of three compartments: susceptible, infective or recovered (and hence immune). The transmission rate to group ai from group bj was given by $\beta_{ai,bj} = qm_{ai,bj}/P_{ai}$, where q was a scaling factor dependent on the basic reproduction number [4]. The final epidemic size in each age group a and contact class i, ϕ_{ai}, could therefore be found by solving the following coupled equation [6],

$$\phi_{ai} = 1 - \exp\left(-\sum_{b=1}^{A}\sum_{j=1}^{C} \beta_{ai,bj} P_{bj} \phi_{bj} \right) . \tag{S3}$$

Estimating reported contacts between age groups

When constructing the model, we divided the participants into A groups of equal size (Fig. S3). However, as A varied, the age groups used did not always line up with the three boundaries in the survey (age under 20, 20 to 65, over 65). We therefore had to estimate $m_{a,b}$, the mean number of contacts in group a reported by individuals in group b, using contact data from the Hong Kong survey and population age distribution data from the 2011 census [1]. First we used census data [1] to calculate $K_{i,x}$, the proportion of each age group i that fall within age boundary B_x, where

$$B_x = \begin{cases} [0, 20] & \text{if } x = 1; \\ (20, 65] & \text{if } x = 2; \\ (65, 105] & \text{if } x = 3 . \end{cases}$$

Next, we used $K_{i,x}$ to estimate the number of contacts between age groups by assuming that individuals make contacts at random, based on their reported contacts and total available contacts in the population. Suppose there are N people in a population and n total contacts, with degree distribution $\{Q_k\}_{k=1}^{n}$. If an individual makes a contact at random, the probability of making a contact with a person who has j total contacts is:

$$\mathbb{P}(\text{meet person with } j \text{ contacts}) = \frac{jQ_jN}{n} = \frac{jQ_jN}{N\sum_{k=1}^{\infty} kQ_k} = \frac{jQ_j}{\sum_{k=1}^{\infty} kQ_k} .$$

We extended this approach to find the expect number of contacts reported by individuals in age group b that are in age group a. If P_a denotes the proportion of individuals in age group a in the 2011 census [1], where $\sum_{a=1}^{A} P_a = 1$, we have

$$m_{a,b} = \sum_{x=1}^{3}\sum_{y=1}^{3} K_{b,y} m_{x,b} \frac{m_{y,a} K_{a,x} P_a}{\sum_{k=1}^{A} m_{y,k} K_{k,x} P_k} \ . \tag{S4}$$

The expression for $m_{a,b}$, or $m_{a,bj}$ if age group b has been divided into multiple contact classes, can then be used with Equation S2 to derive the transmission rates that appear in Equation 1.

Example: Estimating reported contacts between age groups

Here, we show how to estimate $m_{a,b}$, the mean number of contacts in group a reported by individuals in group b, using contact data from the 2009/10 Hong Kong serological survey and population age distribution data from the 2011 census[1]. Suppose we assume twelve age groups in the model (i.e. $A = 12$). The second age group, which for now we denote b, contains individuals with ages ranging from 15.5 to 21.2, and the penultimate age group, a, contains people of ages 60.7 to 67.4 (Figure S3). According to the population age distribution:

$$K_b = \{0.783, 0.217, 0\} \ \text{and} \ K_a = \{0, 0.735, 0.265\} \ . \tag{S5}$$

Based on the Hong Kong survey data, individuals in group b had an average of 6.66 contacts with 20–65 year olds and 0.10 contacts with over 65s. In addition, individuals in group a had an average of 0.41 contacts with under 20s and 12.77 contacts with 20–65 year olds. Suppose we want to estimate how many contacts reported by under 20s in group b are with under 65s in group a. Using Equation S4, we have

$$m_{2,b} \frac{m_{1,a} K_{a,2} P_a}{\sum_{k=1}^{A} m_{1,k} K_{k,2} P_k} = 0.05 \ . \tag{S6}$$

We can find the total contacts from age group b to age group a by summing up all possible combinations, weighting each by the relevant term in K_b, as in Equation S4. This gives $m_{a,b} = 0.15$.

Comparison with network model

When there is only one age group, and R_0 is small (but greater than one), the model formulation is similar to that of a network approach. The attack rate in age age group a and contact class i is given by

$$\phi_{ai} = 1 - \exp\left(-\sum_{b=1}^{A}\sum_{j=1}^{C}\beta_{ai,bj}P_{bj}\phi_{bj}\right) . \tag{S7}$$

When there are several contact classes, but only one age group, and q is small this can be expressed as

$$\phi_i = 1 - \exp\left(-\frac{q\sum_{j=1}^{C} m_j P_j \phi_j m_i}{\sum_{j=1}^{C} m_j P_j}\right) \tag{S8}$$

$$\approx 1 - \left(1 - q\left[1 - \frac{\sum_{j=1}^{C} m_j P_j (1-\phi_j)}{\sum_{j=1}^{C} m_j P_j}\right]\right)^{m_i} \tag{S9}$$

where m_i is the mean number of contacts reported by individuals in class i. This equation has the same form as the percolation approximation for final epidemic size in a network model[5], though it does not account for reduction in available susceptible contacts as a result of network structure.

Simulation study to test robustness of model identification (Figure S1)

We used a simulation study to test whether our model could correctly identify the 'true' model among a range of candidate models. First we simulated attack rates for a specific number of age and contact classes and contact type, finding ϕ_{ai} for each age-contact group using Equation 1. For each participant in a given age-contact group ϕ_{ai}, we then simulated infection data from a Bernouilli distribution with probability $1 - \phi_{ai}$. Given this simulated data, we repeated the analysis in Figure 3, calculating the AIC for each candidate model in the framework, and using ΔAIC to identify the one with most support of those tested. Results are shown in Figure S1.

Sensitivity of results to inclusion of small background risk (Figure S2)

Some of the models with multiple contact classes in Figure 3B had classes consisting solely of individuals – some of whom had been infected – that had no reported close contacts. The

likelihood of such people seeing infection given the model assumptions was therefore zero. To assess whether our results were sensitive to these assumptions, we considered a framework with an additional small background rate of random contact among all members of the population. When this background risk is included, Equation 1 becomes

$$\phi_{ai} = 1 - \exp\left(-\sum_{b=1}^{A}\sum_{j=1}^{C}\beta_{ai,bj}P_{bj}\phi_{bj}\right) + h \tag{S10}$$

where h is a parameter to be found. Results are shown in Figure S2.

Relatively susceptibility in older groups

In Equation 1, the final size of an epidemic in each group was calculated as

$$\phi_{ai} = 1 - S_{ai}(\infty)/S_{ai}(0) \ ,$$

where $S_{ai}(0) = N_{ai}$ denotes the number of individuals in group ai that are susceptible to infection at the start the epidemic and $S_{ai}(\infty)$ denotes that number that remain susceptible – and hence uninfected – at the end. To include relatively susceptibility in age groups older than δ, we solve Equation 1 as before, but with $S_{ai}(0) = \alpha N_{ai} \leq N_{ai}$. Specifically, we define the level of susceptibility at the start of the epidemic as

$$S_{ai}(0) = \begin{cases} N_{ai} & \text{if youngest person in age group } a \text{ is under age } \delta; \\ \alpha N_{ai} & \text{else .} \end{cases}$$

Bootstrap resampling of data (Figure S4)

Our model made the assumption that social contacts in our sample are representative of the population. To test the sensitivity of results in Figure 5B, we therefore repeated our analysis with different datasets. As we did not have additional empirical data, we instead used bootstrap samples: we obtained a new dataset of 762 individuals by resampling the 762 individuals in the Hong Kong dataset with replacement. The probability a specific participant is included in the resampled data set is $1 - (1 - 1/n)^n$. For large data sets – like the Hong Kong study – this can

be approximated by $1 - 1/e \approx 0.63$. We then ran the analysis for Figure 5B for ten different resampled datasets. For each set of data, we found the AIC for each number of age groups, then calculated the values of ΔAIC and plotted the relationship between model resolution and performance (Figure S4).

References

[1] Demographic Statistics Section (2011) Hong kong population census. Census and Statistics Department .

[2] Mossong J, Hens N, Jit M, Beutels P, Auranen K, et al. (2008) Social contacts and mixing patterns relevant to the spread of infectious diseases. PLoS Med 5: e74.

[3] Riley S, Kwok KO, Wu KM, Ning DY, Cowling BJ, et al. (2011) Epidemiological characteristics of 2009 (H1N1) pandemic influenza based on paired sera from a longitudinal community cohort study. PLoS Med 8: e1000442.

[4] Wallinga J, Teunis P, Kretzschmar M (2006) Using data on social contacts to estimate age-specific transmission parameters for respiratory-spread infectious agents. American Journal of Epidemiology 164: 936.

[5] Meyers LA, Pourbohloul B, Newman MEJ, Skowronski DM, Brunham RC (2005) Network theory and sars: predicting outbreak diversity. J Theor Biol 232: 71-81.

[6] Riley S, Wu JT, Leung GM (2007) Optimizing the dose of pre-pandemic influenza vaccines to reduce the infection attack rate. PLoS Med 4: e218.
